# Isothiazolinones in Disposable Rubber Gloves—Results of Chemical Analysis

**DOI:** 10.1111/cod.70087

**Published:** 2026-01-15

**Authors:** Katri Suuronen, Katriina Ylinen, Sari Suomela, Maria Pesonen

**Affiliations:** ^1^ Occupational Medicine Finnish Institute of Occupational Health Helsinki Finland; ^2^ Work Environment Services Finnish Institute of Occupational Health Helsinki Finland

**Keywords:** benzisothiazolinone, BIT, chloromethylisothiazolinone/methylisothiazolinone, CMI/MI, hand dermatitis, methylisothiazolinone, MI, octylisothiazolionone, OIT, preservative

## Abstract

**Background:**

Contact allergy to benzisothiazolinone (BIT) has increased in recent years, but exposure to it is not always found. It's important to know whether the gloves might be an isothiazolinone source especially in patients with symptoms from gloves and isothiazolinone contact allergy with or without allergy to rubber chemicals.

**Objectives:**

To present results of chemical analysis of isothiazolinones in disposable rubber gloves of patients in an occupational dermatology clinic.

**Methods:**

We went through our chemical analysis record from 2018–2025 and identified isothiazolinone analyses done from disposable rubber gloves. The chemical analysis of glove extracts was done by liquid chromatography‐mass spectrometry (LC–MS). We screened the respective patients' files and collected information on their occupation, glove usage, patch test reactions as well as basic information on their hand eczema.

**Results:**

We discovered BIT in 30/54 (60%) analysed gloves (27 nitrile rubber gloves, 2 neoprene rubber gloves and 1 natural rubber glove) in concentrations of 0.3–73.7 ppm (mean 12.7 ppm, median 4.2 ppm). Methylisothiazolinone (MI) was found in solitary gloves in small concentrations. Isothiazolinone‐containing gloves were samples from altogether 21 patients, and six of them had several gloves that contained isothiazolinones. Many patients had also other isothiazolinone sources at work or at home.

**Conclusions:**

Disposable rubber gloves are a possible BIT source. Several gloves contained BIT in a concentration that may be enough to induce contact allergy when gloves are used frequently.

## Graphical abstract

Chemical analysis revealed benzisothiazolinone (BIT) in 30/54 disposable rubber gloves (27 nitrile rubber gloves, 2 neoprene rubber gloves and 1 natural rubber glove) in concentrations of 0.3–73.7 ppm. Methylisothiazolinone (MI) was found in six nitrile rubber gloves in small concentrations.

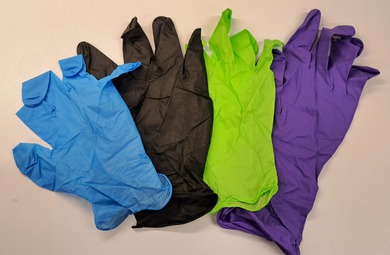

## 1 Introduction

Isothiazolinones are widely used as preservatives in water‐containing cosmetics, industrial products and occupational and household detergents. Contact allergy (CA) to methylisothiazolinone (MI) seems to be declining in Europe largely because of regulatory restrictions concerning cosmetics, whereas CA to benzisothiazolinone (BIT) that is not allowed in cosmetics has increased in recent years [1, 2]. Concerns have been raised as regards the use of isothiazolinones in non‐cosmetic products which may act as hidden allergen sources [3]. Liden and White [4] also pointed out recently that the relevance of isothiazolinone allergy, especially to BIT, is often difficult to show due to lacking information on exposure.

In our occupational dermatology clinic, we usually consider products such as industrial and household detergents, water‐borne paints and personal hygiene products (in cases of MI and chloro‐methyl−/methylisothiazolinone (CMI/MI)), as the most likely causatives of isothiazolinone CA [5]. However, we also see patients with hand dermatitis, isothiazolinone CA (with or without rubber CA's) and frequent glove use, which has raised the question of whether their gloves might contain isothiazolinones that could have played a role in their disease. In such cases, we have analysed the patients' gloves for isothiazolinones. Here we present our findings of these investigations.

## 2 Methods

### 2.1 Collection of Data

At the Finnish Institute of Occupational Health (FIOH), all dermatology patients are examined for suspected occupational skin disease and they come from all over Finland. We have a chemical laboratory within the institute where we can send our patients' materials for chemical analysis if allergies are revealed in patch tests and we cannot find enough information on exposure by other means. We keep records of the chemical analyses of patient samples.

We screened the chemical analysis record from 1/2018 to 5/2025 to identify isothiazolinone analyses from patients' gloves for the present study. We then collected the respective patients' clinical data: age, sex, occupation, patch test reactions to gloves, isothiazolinones and rubber chemicals, other identified isothiazolinone sources at work and at home, location of dermatitis and diagnoses.

### 2.2 Patch Testing

The patients were tested with a modified Finnish baseline series and an in‐house series of antimicrobials, with other series according to their occupational exposure including the rubber series and with their own materials such as protective gloves and chemicals from the workplace. The baseline series contained the following rubber chemicals: thiuram mix, thiourea mix, black rubber mix, mercapto mix and mercaptobenzothiazole. The rubber series includes the constituents of the rubber mixes tested in the baseline series. BIT (CAS 2634‐33‐5) was tested in 0.05% petrolatum (pet.) until 10/2018 and after that in 0.1% pet. MI (CAS 2682‐20‐4) was tested in 0.2% aqua, (CMI/MI, CAS 55965‐84‐9) in 0.02% aqua, octylisothiazolinone (OIT, CAS 26530‐20‐1) in 0.1% pet., and dichloroctylisothiazolinone (DCOIT, CAS 64359‐81‐5) in 0.1% pet. Patch test substances for BIT, MI, CMI/MI and OIT were purchased from Chemotechnique, and that of DCOIT was prepared in‐house from DCOIT purchased from Chemos GmbH. Patients' own rubber gloves, when available for patch testing, were tested as is, moistened with acetone and with aqua. Some patients were also tested with ultrasonic extracts (UE) in acetone prepared according to Bruze et al. [6]. In solitary cases, we tested the gloves also moistened with ethanol or as UE in ethanol (see Table 1). In some cases, we asked the patients after patch testing to send additional glove samples for chemical isothiazolinone analyses. In such cases, it was not feasible to patch test the glove samples in the respective patients.

**TABLE 1:** Concentration of benzisothiazolinone (BIT) and methylisothiazolinone (MI) in 31 disposable protective gloves in 2018–2025 (in descending order of BIT concentration); the respective patient's occupation and patch test reactions to gloves, isothiazolinones and rubber chemicals.

Glove name	Manufacturer or distributor	Year of analysis	BIT in the glove, % w/w (ppm)	MI in the glove % w/w (ppm)	Patient's patch test reactions to BIT (0.1% pet), MI (0.2% aq) and CMI/MI (0.02% aq)	Patient's patch test reactions to the glove^a^	Exposure to isothiazolinones via products other than gloves	CA to rubber chemicals (yes/no)	Patient's occupation	Patient no.
Nitrile gloves^b^										
Tegera 858 Nitrile gloves (accelerator free, lilac)	Ejendals AB	2023	0.00737 (73.7)	0.00166 (16.6)	BIT+ MI+++ CMI/MI++	As is: neg UE/acet: +	BIT‐ and MI‐containing detergents at work	Yes	Farm relief worker	1
Tegera 858 Nitrile gloves (accelerator free, lilac)	Ejendals AB	2025	0.00655 (65.5)	0.001 (10)	BIT neg MI++ CMI/MI++^c^	As is: neg	MI in detergents at work	No	Farm relief worker	2
Tegera 858 Nitrile gloves (accelerator free, lilac)	Ejendals AB	2025	0.00541 (54.1)	0.00067 (6.7)	BIT+++ MI+++ CMI/MI+++^c^	As is/aq: ++ As is/acet: ++ UE/acet: +	“Isothiazolinone” in a shower gel (personal report by patient)	No	Farmer	3
Tegera 858 Nitrile gloves (accelerator free, lilac)	Ejendals AB	2025	0.00493 (49.3)	0.00124 (12.4)	BIT neg MI+++ CMI/MI++^c^	NT	MI in dishwashing detergent at work and home	Yes	Farmer	4
KI‐SAL Nitrile Glove (blue)	KI‐SAL Oy/ Topglove Corp.	2022	0.0030 (30)	< 0.00002	BIT++ MI++ CMI/MI?+	As is: neg UE/acet: +	MI and CMI/MI in detergents at work and home	Yes	Baker	5
Hygostar Safe super	Franz Mensch Gmbh	2025	0.00205 (20.5)	< 0.00003	BIT neg MI+++ CMI/MI++^c^	NT	MI in dishwashing detergent at work and home	Yes	Farmer	4
Hygostar Safe Super (accelerator free)	Franz Mensch Gmbh	2023	0.0018 (18)	< 0.00005	BIT++ MI++ CMI/MI?+	NT	MI and CMI/MI in detergents at work and home	Yes	Baker	5
Evercare Nitrile Lime accelerator free gloves	Evercare Medical	2025	0.00145 (14.5)	< 0.00005	BIT neg MI neg CMI/MI neg^d^	As is: neg	Not found	Yes	Medical laboratory technician	6
Showa Chemrest	SHOWA Best Glove Inc.	2025	0.00105 (10.5)	0.00114 (11.4)	BIT neg MI+++ CMI/MI++^c^	NT	MI in dishwashing detergent at work and home	Yes	Farmer	4
Alphatec 79–700 Nitrile (blue)	Ansell Gmbh	2025	0.00081 (8.1)	< 0.00003	BIT neg MI++ CMI/MI++	As is: neg	MI in detergents at work	No	Farm relief worker	2
Disposable nitrile grip glove (orange)	Würth Industrie Service Gmbh	2023	0.00069 (6.9)	0.0008 (8)	BIT+ MI+++ CMI/MI++	As is: neg.	BIT‐ and MI‐containing detergents at work	Yes	Farm relief worker	1
Biltema powder free nitrile gloves (black)	Biltema Oy	2024	0.00061 (6.1)	< 0.00003	BIT+ MI?+ CMI/MI++	As is: neg	CMI/MI in hand cleanser/liquid soap at workplace	No	Mechanic	7
Selefa Sense Nitrile (black)	Evercare Medical (OneMed Group Oy)	2024	0.00057 (5.7)	0.0017 (17)	BIT neg MI neg CMI/MI?+	NT	CMI/MI and MI in indoor paints and putties	No	Painter	8
Hygostar Powergrip nitrile (orange)	Weidinger Gmbh	2024	0.00053 (5.3)	< 0.00005	BIT neg MI?+ CMI/MI+++	As is: neg	Not found	No	Laboratory technician	9
Kingfa Nitrile examination gloves	KINGFA Medical Sci.& Tech. Co. Ltd	2024	0.00046 (4.6)	< 0.00005	BIT neg MI+++ CMI/MI+++	As is: neg UE/acet: neg UE/alc: neg	MI‐, CMI/MI‐, BIT‐ and OIT‐containing detergents at work and at home	Yes	Assistant nurse	10
Abena Nitrile	Abena A/S	2025	0.00038 (3.8)	< 0.00003	BIT neg MI+++ CMI/MI+++^c^	As is: neg UE/acet: neg	MI‐containing detergents at home	No	Laboratory technician	11
Diamante Nitrile examination Gloves	Global Food Hygiene (unverified information)	2025	0.00035 (3.5)	< 0.00003	BIT neg MI++ CMI/MI++^c^	NT	MI‐containing shampoo, previously used at home (personal report by the patient)	No	Assistant nurse	12
Nitrile Examination Gloves (light blue)	Unknown manufacturer	2025	0.00034 (3.4)	< 0.00003	BIT neg MI++ CMI/MI++^c^	NT	MI‐containing shampoo, previously used at home (personal report by the patient)	No	Assistant nurse	12
Kingfa Medical nitrile	KINGFA Medical Sci.& Tech. Co. Ltd	2024	0.00032 (3.2)	< 0.00005	BIT neg MI++ CMI/MI+++	As is: neg UE/acet: neg	MI in dishwashing liquid at work	No	Assistant nurse	13
Kingfa Nitrile (black)	KINGFA Medical Sci.& Tech. Co. Ltd.	2024	0.00031 (3.1)	< 0.00005	BIT neg MI++ CMI/MI++	As is: neg	MI in a previous workplace detergent	No	Kitchen worker	14
Black, nitrile powder‐free disposable glove	Würth Industrie Service Gmbh	2025	0.00026 (2.6)	< 0.00005	BIT++ MI neg CMI/MI neg	As is: neg	BIT in metalworking fluid	No	Machinist	15
Bea Pro Nitrile	Berner Oy	2024	0.00024 (2.4)	< 0.00005	BIT+ MI neg CMI/MI neg	NT	BIT‐containing detergent at work	No	Dental technician	16
Brücke nitrile disposable black	Tokmanni Oy	2025	0.00019 (1.9)	< 0.00005	BIT neg MI++ CMI/MI++	As is: neg	Not found	No	Cleaner	17
Abena Classic Sensitive Nitrile	Abena A/S	2024	0.00018 (1.8)	< 0.00005	BIT neg MI+++ CMI/MI+++	As is/aq: ?+ UE/alc: ?+	MI‐, CMI/MI‐, BIT‐ and OIT‐containing detergents at work and at home	Yes	Assistant nurse	10
Nitras 8300 Supreme Nitrile (accelerator free)	Nitras Safety	2025	0.00016 (1.6)	< 0.00005	BIT neg MI+++ CMI/MI++^c^	As is?+	MI in dishwashing detergent at work and home	Yes	Farmer	4
Worksafe Examination Gloves Blue Nitrile	Worksafe, Singapore	2025	0.00015 (1.5)	< 0.00003	BIT neg MI++ CMI/MI++^c^	NT	MI‐containing shampoo, previously used at home (personal report by the patient)	No	Assistant nurse	12
Touchntuff NBR	Ansell Gmbh	2022	0.00003 (0.3)	< 0.00002	BIT neg MI?+ CMI/MI+	Session I: As is: neg UE/acet: ?+/IR Session II: As is: neg UE/acet: +	MI in dishwashing liquid at home	Yes	Laboratory technician	18
Mooni Disposable Nitrile Gloves	Mooni Ltd	2023	< 0.00004	0.0013 (13)	BIT neg MI neg CMI/MI++	As is/acet: ?+ As is/alc: ?+ UE/acet++	CMI/MI‐containing detergents and personal hygiene products at home	No	Assistant nurse	19
Neoprene gloves										
Microflex neotouch	Ansell Ltd.	2021	0.00073 (7.3)	< 0.00005	BIT neg MI?+ CMI/MI+	As is/acet: + As is/aq: ?+ UE/acet: ++ UE/alc: ++	BIT‐containing dishwashing liquid at work, CMI/MI in personal hygiene products at home	No	Laboratory technician	20
Tegera Neoprene gloves	Ejendals AB	2023	0.00021 (2.1)	< 0.00004	BIT+ MI+++ CMI/MI++	As is: neg UE/acet: +	BIT‐ and MI‐containing detergents at work	Yes	Farm relief worker	1
Natural rubber latex gloves										
Mercator Dermagel Coated	Mercator Medical S.A.	2024	0.00015 (1.5)	< 0.00005	BIT neg MI neg CMI/MI+	As is: neg UE/acet: neg	Not found	No	Dental nurse	21

*Note:* Of a total of 21 patients, six (patients No. 1, 2, 4, 5, 10 and 12) had more than one glove sample analysed and shown to contain isothiazolinones, therefore these patients appear on more than one row.

Abbreviations: acet, acetone; alc, ethanol; aq, aqua; BIT, benzisothiazolinone; CMI/MI, chloromethyl/methylisothiazolinone; MI, methylisothiazolinone; neg, negative test; NT, not tested; pet, petrolatum; UE, ultrasonic extract.

^a^ Most gloves were tested as is/aqua and as is/acetone; some gloves were tested also as ultrasonic extracts in acetone and/or ethanol.

^b^ Twenty‐eight individual gloves representing 24 products (one glove model was used by four patients).

^c^ Patient had positive patch test reactions also to octylisothiazolinone (OIT) and/or dichloro‐octylisothiazolinone (DCOIT).

^d^ Patent had a doubtful reaction to DCOIT.

### 2.3 Chemical Analysis of Isothiazolinones in Gloves

Material samples were cut from the fingers or palm of the patients' gloves and accurately weighed, the amount of sample was approximately 200 mg. The samples were extracted into 5 mL of methanol/water (1:3) mixture in an ultrasonic bath for 15 min and overnight at room temperature. After extraction, the samples were filtered by polytetrafluoroethylene (PTFE) membrane syringe filters (0.22 μm pore size). The compounds were analysed by liquid chromatography using Thermo TSQ Quantum Ultra triple quadrupole mass spectrometry (LC–MS/MS) applying multiple reaction monitoring (MRM) method. The compounds detected until November 2022 were MI, CMI (CAS 26172–55‐4), OIT and BIT. DCOIT was added to the method after that. The analytes were separated on a Waters Symmetry Shield C18 column (3.5 μm, 2 × 150 mm) and ionised with electrospray ionisation (ESI). The ions detected were m/z 116 and 99 (MI), m/z 150 and 87 (CMI), m/z 152 and 109 (BIT), m/z 214 and 102 (OIT), m/z 282 and 170 (DCOIT). The mobile phase eluent A was water 95%/methanol 5%/formic Acid 0.5% and eluent B was water 5%/methanol 95%/formic Acid 0.5%. Flowrate was 0.2 mL/min and injection volume 10 μL. The limit of determination was approx. 0.1 μg/sample.

Quantification was carried out using pure reference substances purchased from Sigma Aldrich, except for a technical grade Kathon CG which was used as a standard for CMI/MI before year 2023; after that we used certified reference standards (CRM, PHR1597) from Supelco Sigma Aldrich.

## 3 Results

During 2018–May 2025, we analysed a total of 52 disposable rubber gloves (41 nitrile rubber, 8 neoprene rubber and 3 natural rubber latex gloves) for their isothiazolinone content from patients who all had hand eczema and showed positive or doubtful patch test reactions to at least one of the isothiazolinones tested. We discovered BIT in 30 (60%) of them (27 nitrile rubber gloves, 2 neoprene rubber gloves and 1 natural rubber glove) in concentrations of 0.3–73.7 ppm (median 4.2 ppm, average 12.7 ppm). MI was discovered in six nitrile gloves (13%), the concentration being 0.8–16.6 ppm (median 8.4 ppm, average 8.1 ppm). None of the gloves contained OIT, DCOIT or CMI (Table 1). One of the analysed glove models, Tegera 858, which was used by four farm workers, was found to contain high amounts of BIT and MI.

Isothiazolinone‐containing gloves were samples of altogether 21 patients, six patients having several gloves that contained isothiazolinones (Table 1). Many patients had also other isothiazolinone sources at work or at home. In four patients, workplace gloves were the only source of isothiazolinone exposure we were able to identify. Six patients with BIT CA had used gloves that contained BIT. Five patients had CA to MI and gloves that contained MI. Of these patients, three were allergic to both BIT and MI and their gloves (Tegera 858) also contained both. Most of our patients had a lengthy history of hand eczema: 19 of 21 patients reported recurring or chronic hand eczema for at least 1 year, nine had a history of hand eczema for ≥ 10 years and only two patients' hand eczema had been transient and of short duration.

Seventeen out of 31 gloves that contained BIT or MI were patch‐tested on the respective patient as is and/or as UE: 6/17 gave at least one positive reaction, some induced uncertain or irritant reactions. Isothiazolinones did not explain all the positive glove reactions, as in some cases the patient was allergic to rubber chemicals or to another isothiazolinone than that detected in the chemical analysis. The strongest patch test reaction to glove material (++ tested as is and as UE in acetone) was produced by a glove batch (Tegera 858) that contained 0.0054% (54 ppm) BIT and 0.00067% (6.7 ppm) MI on a patient that was strongly allergic to both (patient 3, Table 1).

In addition to CA to isothiazolinones, six patients had CA to thiuram‐ or dithiocarbamate rubber chemicals and three of them reacted to their own gloves. Two patients (19 and 20) had patch test reactions to their own gloves which were not explained by their isothiazolinone allergies and they were not allergic to rubber chemicals either. These glove reactions were probably due to other, unknown glove ingredients which we were unable to identify.

## 4 Discussion

We present our findings on isothiazolinones in disposable rubber gloves from patients with isothiazolinone CA. Our chemical analyses show that BIT was quite commonly detected in the gloves and that MI was occasionally found, too. The concentrations were mostly small, but in some cases, we discovered noteworthy concentrations of BIT, the highest concentration being 75 ppm. BIT was also found in accelerator‐free gloves, which indicates that accelerator‐free gloves are not free from all allergens.

Liden et al. pointed out recently that isothiazolinone exposure sources other than cosmetics are increasing, and that the relevance of BIT allergy is often difficult to establish mostly due to the lack of information on exposure [3, 4]. The observation was based on data on imported and manufactured biocides, which are relevant to chemicals and detergents but not to gloves that are imported to Europe as ready‐made products. In our patients, as part of the examinations of suspected occupational contact dermatitis, we thoroughly search for the possible sources of exposure to the contact allergens detected. In most of the patients reported herein we also found other sources of isothiazolinone exposure than the gloves. However, based on our present results, disposable nitrile rubber gloves and possibly other rubber gloves are indeed another possible source of exposure to BIT and also to MI. Considering the intensive use of disposable rubber gloves in many occupations, a substantial population might be at risk of developing BIT CA from gloves. Of note, BIT and OIT were recently found in clothing, textile or footwear articles on the Italian market [7] indicating that isothiazolinone sources are versatile.

Information about preservatives in gloves is very difficult to find [8]. Batches of the same glove model might differ in their composition. BIT in rubber gloves most likely originates from preserving the main raw material in its liquid form, i.e., the synthetic or natural rubber latex. According to a United States standard [9], BIT is allowed as a biocide in uncured rubber latexes (synthetic and natural) for producing gloves for foodstuff contact; the maximum allowed concentration is 0.02% (200 ppm). According to our discussions with a representative of rubber glove industry (Dr. Pierre Hoerner, Ineo Tech Sdn Bhd, Malaysia; e‐mail communications in 2024), this kind of preservation is especially used for nitrile rubber latexes; MI is not mentioned in the standard but when present, it has most likely been used for the same purpose.

It is quite difficult to estimate what isothiazolinone concentration in a glove is high enough to induce CA. All our patients had hand eczema, and most of them used gloves frequently and thereby had repeated skin contacts with isothiazolinones via them. In earlier reports from our clinic, BIT was analysed in PVC gloves in 9–32 ppm; it was estimated that 20 ppm might be enough to induce CA in patients with preexisting hand eczema [10, 11]. In the present study, many of the rubber gloves that contained BIT had similar or even higher concentrations than those previously reported in PVC gloves. Recently, BIT was analysed in 20 PVC gloves in the United States and found in all of them [12], whereas MI was found in reusable nitrile rubber gloves [13].

In most of our patients, their gloves were not the only source of isothiazolinones. Many of them had other plausible causes of their hand eczema than isothiazolinone CA, such as CA to rubber chemicals, which was the case in 29% of the patients. We therefore do not consider isothiazolinone‐containing gloves as the only possible cause of sensitization to isothiazolinones nor the sole cause of hand eczema in our patients. However, as long duration of hand eczema was common in our patients, it seems plausible that in at least some patients, the pre‐existing hand eczema might have facilitated sensitization to isothiazolinones when isothiazolinone‐containing gloves were used. Oftentimes, the patients used the gloves for prolonged times during their work tasks.

At FIOH, we test nearly all patients' rubber gloves as is (moistened with acetone and aqua) and if the patient has symptoms specifically related to gloves, also as UE's in acetone according to Bruze et al. [6]. In suspected CA to a glove material, patch testing of UE both in acetone, aqua and ethanol is recommended (personal communication with professor Magnus Bruze in 2025). Investigations of occupational contact dermatitis often require extensive patch testing of workplace materials, and there is not always enough space for testing all our patients' gloves in three different UE's. When testing glove materials as is, we cut a glove piece to fit an ordinary Finn Chamber with 0.8 mm diameter. In case of UE, we apply 15 μL of the extract (‘stock solution’ after evaporating the extract to dryness and dilution of the residue to 1 mL solvent) to a Finn Chamber once. In both cases, the amount of isothiazolinones might be too small to induce a test reaction. We are currently considering ways to enhance exposure to substances present in glove materials in our patch tests in the future; this could be done, for example, by increasing the size of the glove pieces as has been done in some clinics before [14, 15, 16] and/or applying several 15‐μL doses of a glove extract (stock solution) in one chamber with evaporation of the solvent after each dose. Some authors have suggested glove repeated application test (GRAT), similar to repeated open application test (ROAT) as a means to increase the possibility of positive skin tests with patient's own gloves [17].

It is important to realise that despite extensive efforts, gloves may remain negative in patch testing even if they contained relevant allergens and were a probable cause of the patients' skin symptoms. In patients with isothiazolinone contact allergy, the relevance of gloves should be evaluated based on not only patch test results but also on chemical analyses of the gloves, if possible.

As a limitation, our small sample set was collected from highly selected patients with isothiazolinone CA and symptoms related to disposable gloves. Nevertheless, disposable rubber gloves seem to contain BIT quite often. For the time being, specific analysis of each patient's gloves may be needed to show exposure to isothiazolinones through glove usage. A large market study on isothiazolinones in disposable rubber gloves would be helpful for the clinical work.

## 5 Conclusions

We conclude that disposable rubber gloves, especially nitrile rubber gloves, are a possible source of exposure to BIT. MI was also detected in some rubber gloves. The detected concentrations may be high enough to elicit allergic contact dermatitis in individuals with BIT contact allergy, and possibly also to sensitise if the gloves are used frequently.

## Author Contributions

**Katri Suuronen:** conceptualization, investigation, writing – original draft, methodology, writing – review and editing, data curation. **Sari Suomela:** conceptualization, investigation, writing – review and editing, writing – original draft, methodology, data curation. **Maria Pesonen:** conceptualization, investigation, writing – original draft, writing – review and editing, methodology, data curation.

## Funding

The authors have nothing to report.

## Conflicts of Interest

The authors declare no conflicts of interest.

## Data Availability

The data that support the findings of this study are available on request from the corresponding author. The data are not publicly available due to privacy or ethical restrictions.
